# Laser-Induced Graphene-Based Wearable Epidermal Ion-Selective Sensors for Noninvasive Multiplexed Sweat Analysis

**DOI:** 10.3390/bios12060397

**Published:** 2022-06-09

**Authors:** Jianjun Liao, Xiangya Zhang, Zihan Sun, Hande Chen, Jian Fu, Hewei Si, Chengjun Ge, Shiwei Lin

**Affiliations:** 1 Key Laboratory of Agro-Forestry Environmental Processes and Ecological Regulation of Hainan Province, School of Ecological and Environmental Sciences, Hainan University, Haikou 570228, China; liaojianjun008@hainu.edu.cn (J.L.); xiangya94264@gmail.com (X.Z.); zihansun266@gmail.com (Z.S.); 990963@hainanu.edu.cn (C.G.); 2 Hainan Unican Science and Technology Innovation Institute, Haikou 571152, China; chenhande53@gmail.com (H.C.); fujian199702@gmail.com (J.F.); 3 School of Materials Science and Engineering, Hainan University, Haikou 570228, China; 19080500110011@hainanu.edu.cn

**Keywords:** laser-induced graphene, electrochemical sensors, sweat analysis, wearable devices

## Abstract

Wearable sweat sensors are a rapidly rising research area owing to their convenience for personal healthcare and disease diagnosis in a real-time and noninvasive manner. However, the fast and scalable fabrication of flexible electrodes remains a major challenge. Here, we develop a wearable epidermal sensor for multiplexed sweat analysis based on the laser-induced graphene (LIG) technique. This simple and mask-free technique allows the direct manufacturing of graphene electrode patterns on commercial polyimide foils. The resulting LIG devices can simultaneously monitor the pH, Na^+^, and K^+^ levels in sweat with the sensitivities of 51.5 mV/decade (pH), 45.4 mV/decade (Na^+^), and 43.3 mV/decade (K^+^), respectively. Good reproducibility, stability, and selectivity are also observed. On-body testing of the LIG-based sensor integrated with a flexible printed circuit board during stationary cycling demonstrates its capability for real-time sweat analysis. The concentrations of ions can be remotely and wirelessly transmitted to a custom-developed smartphone application during the period in which the sensor user performs physical activities. Owing to the unique advantages of LIG technique, including facile fabrication, mass production, and versatile, more physiological signals (glucose, uric acid, tyrosine, etc.) could be easily expanded into the LIG-based wearable sensors to reflect the health status or clinical needs of individuals.

## 1. Introduction

Wearable sensors are gaining wide attention in personal health monitoring, as they can continuously track health status changes in time for early treatment intervention. With a relatively simple design, the existing wearable sensors are centered on the monitoring of physical and electrophysiological parameters such as heart rate [1], blood pressure [2], electrocardiograms [3], and body motion [4]. Although these are needed, there is an urgent need for the development of wearable devices that can provide biochemical information at a molecular level to retrieve the complete physiological conditions of the wearer.

Sweat contains a wealth of biomarkers related to the metabolites of the human body, including electrolytes (e.g., sodium and potassium), metabolites (e.g., lactate and glucose), and small quantities of hormones and peptides [5,6,7]. The presence of, or variation in, the concentrations of certain biomarkers provides important information on the physiological state [8]. For example, sodium and potassium are indicative of the electrolyte balance and hydration status [9]. Excessive loss of them may lead to hyponatremia, hypokalemia, and muscle cramps [10]. Meanwhile, the sweat of a normal body is slightly acidic [11], and the change in pH in sweat is an indicator of dehydration and muscle fatigue [12]. Importantly, sweat is easily accessible and can avoid conventional invasive blood sampling processes. Therefore, a number of wearable sweat-based sensors, especially wearable electrochemical devices, have recently been developed for continuous noninvasive monitoring of sweat composition [13,14,15,16,17].

Mechanical flexibility and simple array patterning for multiparametric analysis are two critical requirements for wearable electrochemical sensors. Currently, most reported wearable electrochemical sensors are fabricated through conventional microfabrication and screen-printing methods [18,19,20,21,22]. The microfabrication process often needs multistep photolithography, thin-film depositions, and etching, and thus the high manufacturing cost greatly hinders its broad application. The screen-printing method allows the fabrication of sensor patterning directly onto the flexible substrates; however, the complicated ink fabrication, printing resolution, and additional postprocessing printing steps limit its widespread practical applications. Overall, a simple, high-efficient, low-cost fabrication process is highly desired for wearable electrochemical sensors. 

Recently, laser direct writing (LDW) has been widely adopted to fabricate various patterned electrodes through a maskless and vacuum-deposition-free process [23,24,25,26,27]. Utilizing the high-power laser pulses, LDW can convert carbonaceous precursors into highly conductive porous graphene, termed laser-induced graphene (LIG) [24,28]. Moreover, LIG exhibits excellent mechanical flexibility. For example, Kong et al. found the LIG strip still could hold good electrical properties even after 5000 cyclic bending tests [29]. Till now, various substrates, including wood, cloth, food, and paper, have been used to produce graphene layers [30,31,32,33]. Due to these unique properties, LIG has been widely assembled into various wearable electronics for health monitoring [34,35]. 

Herein, a flexible and miniaturized electrode array was fabricated by directing laser engraving polyimide film. The obtained LIG electrodes were subsequently modified with ion-selective membranes to construct wearable multiplexed sensors for simultaneously monitoring the pH, Na^+^, and K^+^ levels in sweat. The wearable sweat sensors showed high sensitivity, good selectivity, and stability. For practical assessment, we designed a signal processing system, which consists of a flexible printed circuit board and a custom-built Android application. Real-time on-body testing was also performed on a human subject and demonstrated its capability for real-time sweat analysis.

## 2. Materials and Methods

### 2.1. Reagents and Materials

Sodium ionophore X, valinomycin (potassium ionophore), polyvinyl chloride (PVC), dioctyl sebacate (DOS), sodium tetraphenylborate (NaTPB), potassium tetrakis (4-chlorophenyl) borate (KTClPB), polyaniline (PANI), poly(3,4-ethylene dioxythiophene):polystyrene sulfonate (PEDOT:PSS). 

### 2.2. Preparation of LIG Electrodes

**Figure 1:**
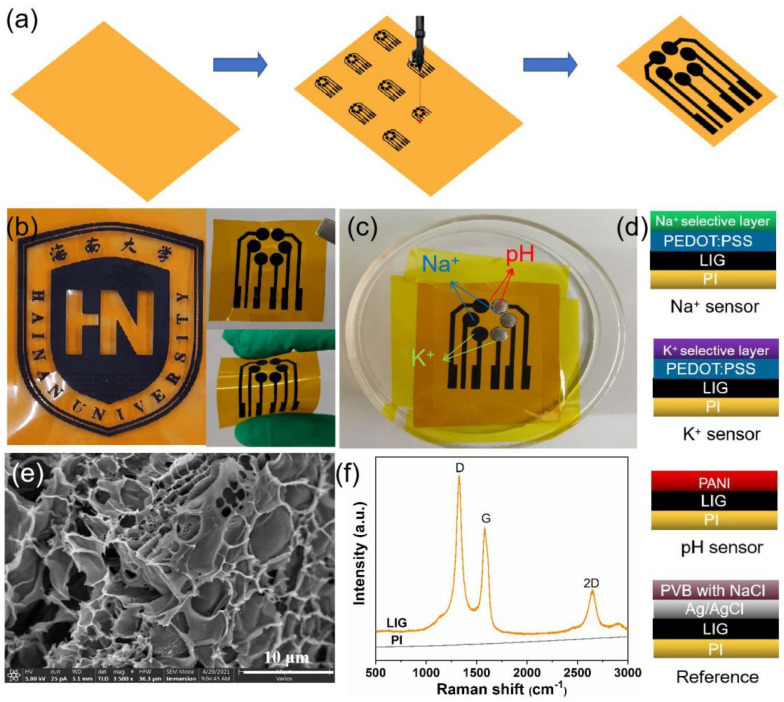
(**a**) Schematic illustration of the fabrication process of LIG electrodes; (**b**) photograph of the logo of Hainan University fabricated by LIG technique, and the photographs of LIG electrodes showing excellent flexibility; (**c**) photograph of a LIG sensor after modification with corresponding ion-selective membranes (black circles, left) and Ag/AgCl ink (silvery circles, right), which could simultaneously detect Na^+^, K^+^, and pH in sweat; (**d**) schematic illustration of the structures of Na^+^, K^+^, pH sensors, and Ag/AgCl reference electrodes; (**e**) SEM image and (**f**) Raman spectra of LIG layer.

The LIG electrodes were prepared via laser-induced carbonization of polyimide (PI) films (75 μm thickness, Kapton^®^) using a commercial CO_2_ laser cutting platform (GCC LaserPro Venus II). As shown in Table S1, the laser power and scanning speed were firstly optimized. The overlarge laser power will burn out the PI film, and the small laser power cannot form LIG pattern on the PI sheet. Therefore, the optimal laser power and scanning speed are set at 60% (Max. 12 W) and 15% (Max. 20 IPS), respectively. CorelDRAW software was used to design the electrode patterns on the PI film surface. The production procedure of LIG electrodes is shown in Figure 1a. The circular region with a 3 mm diameter was used as the working area. The connection wire was passivated with insulation tape to protect it from contact with the sweat or electrolyte. Finally, a strip of copper foil was attached to the end of the wire pad for better electrical connection during measurements. 

### 2.3. Preparation of Na^+^ and K^+^-Selective Sensors

Prior to preparing the sensing electrodes, a PEDOT:PSS solution (Clevios PH500) was dropped onto the sensing area and dried at 120 °C for 1 h. PEDOT:PSS is an excellent ion-to-electron transducer, which can convert the charge carriers from ions to electrons via the doping/de-doping of PEDOT:PSS [36,37]. The Na^+^ ion-selective membrane was prepared by dissolving Na ionophore X, Na-TFPB, PVC, and DOS (weight ratios of 1/0.55/33/65.45) in tetrahydrofuran (1 mL). The K^+^ ion-selective membrane cocktail was prepared by dissolving 2 mg of valinomycin as an ionophore, KTClPB, PVC, and DOS (weight ratios of 2/0.5/32.75/64.75) in 1 mL of cyclohexanone.

### 2.4. Preparation of pH Sensors

For the pH sensor, H^+^ ion-selective electrode was connected with an electrochemical workstation (CHI 660E, Shanghai ChenHua Instruments Co., Shanghai, China) and dipped into a 0.1 M aniline/0.1 M H_2_SO_4_ solution. PANI was electrodeposited using cyclic voltammetry using a potential range varying from −0.2 to 1 V for 25 cycles at 100 mV/s. Commercial Ag/AgCl (1 M KCl) electrode and Pt wire were used as reference and counter electrodes, respectively. Finally, Ag/AgCl paste was drop-coated onto the reference electrode and baked at 120 °C for 5 min. 

### 2.5. Characterization

Surface morphology images were characterized using a field emission-scanning electron microscope (FESEM, Hitachi SU8020, Japan). Raman spectra were acquired using an inVia Raman spectrometer with a 514 nm laser (Renishawin, Wotton-under-Edge, UK). The electrochemical performance was analyzed with an electrochemical analyzer (CHI660E, Shanghai ChenHua Instruments Co., China). Sheet resistance measurements were performed using a four-probe resistance meter (HPS2523, Beijing Jiahang Bochuang Technology Co., Beijing, China).

### 2.6. On-Body Detection

On-body sweat analysis was performed in compliance with the protocol approved by the animal welfare and ethical review board at Hainan University (Issue No. HNUAUCC-2021-00106). The subject (age: 23, female) rode an exercise bike for 60 min to produce sweat. Additionally, the sensor was attached to the forearm of the subject for continuous monitoring of pH, Na^+^, and K^+^ levels in sweat.

## 3. Results and Discussion

As shown in Figure 1a, the patterned LIG sensors can be easily produced by a precise programmable LDW technique. Under the irradiation of a laser beam, the color of PI film changed from light orange to dark, which indicated the formation of graphitic carbon. Meanwhile, a variety of predesigned patterns can also be readily realized, such as the logo of Hainan University shown in Figure 1b, demonstrating the facile, high-efficient, and versatile advantages of the LDW technique. Figure 1e shows the FESEM image of LIG electrodes. The laser-written region exhibited a 3D porous architecture with micrometric holes generated by the emission of gases during the irradiation process [38]. Raman spectrum of the LIG (Figure 1f) further demonstrates the graphitic properties of LIG. There were three characteristic peaks at ~1350 cm^−1^ (D peak), ~1580 cm^−1^ (G peak), and ~2700 cm^−1^ (2D peak), respectively [30,39]. The D peak is related to the formation of sp^2^ carbon bond defects in the graphene. The G peak arises from the C-C bond stretching in graphitic materials and confirms the presence of the sp^2^ carbon networks. The 2D peak is due to the second harmonic of the D band, which further demonstrates the graphene-like features [40,41]. These results indicate that CO_2_ laser irradiation is able to cause carbonization and subsequent graphitization of polyimide. Meanwhile, the obtained LIG electrode (2 × 2 cm) showed a sheet resistance as low as *R*_s_ = 23 Ω sq^−1^ (Table S1), indicating good electrical conductivity. As shown in Figure S1, a LED bulb could be readily lighted when a LIG wire was used as a conductor. Furthermore, after functionalization with various ion-selective membranes (Figure 1c,d), it was revealed that LIG electrodes can be used as a promising platform for wearable sweat-sensing applications. 

**Figure 2:**
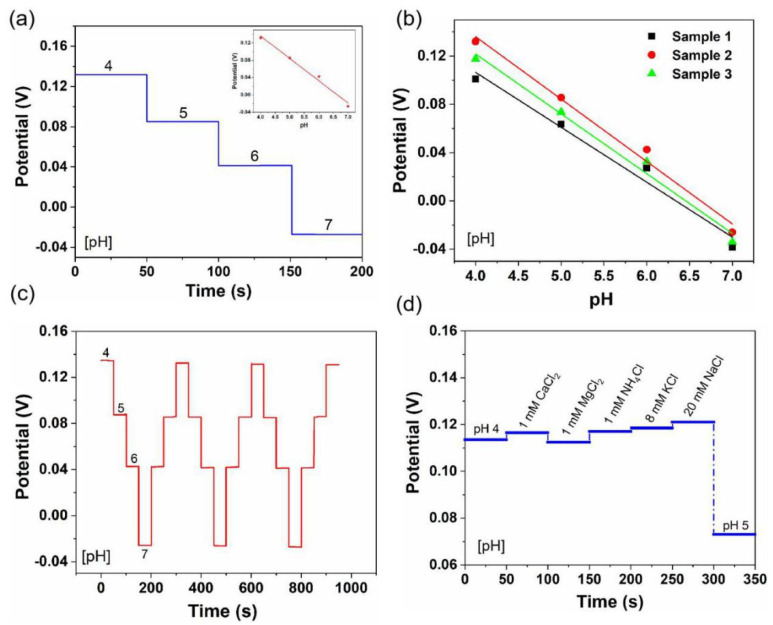
pH-sensing performance of flexible LIG-based sensors: (**a**) sensitivity, (**b**) reproducibility, (**c**) stability, and (**d**) selectivity. Inset in (**a**) is the corresponding calibration plot.

As sweat is found at moderately acidic-to-neutral pH levels, typically between 4.5 and 7.0 [42,43], pH detection was evaluated by measuring the open circuit potential (OCP) value with the variation of pH from 4 to 7 (Figure 2). As shown in Figure 2a, the OCP stably decreased as the pH increased from 4 to 7, and the LIG-based pH sensors exhibited a sensitivity of 51.5 mV/decade, which is close to the Nernst limit of 59.2 mV/decade [44]. Figure 2b shows the reproducibility of three different sensors. The sensitivity showed an insignificant fluctuation from 47.5 to 51.7 mV/decade (RSD = 6.4%). The stability was also measured repeatedly in PBS buffer solution from pH 4 to 7 (Figure 2c). The OCP showed a stable change with the pH level in three complete cycles. The average sensitivity was 51.9 mV/decade (RSD = 0.5%), indicating that LIG-based pH sensors are reproducible and durable. Since sweat contains a variety of electrolytes such as Na^+^, K^+^, H^+^, Ca^2+^, Mg^2+^, and NH_4_^+^, it is essential to examine the selectivity of the wearable sweat sensors. As depicted in Figure 2d, physiologically relevant concentrations of interfering ions (1 mM Ca^2+^, 1 mM Mg^2+^, 1 mM NH_4_^+^, 8 mM K^+^, and 20 mM Na^+^) were added to the PBS buffer solution with pH 4. The change in potential was significantly smaller than the response when the pH value increased to pH 5. This shows that the sensor displays high selectivity.

**Figure 3:**
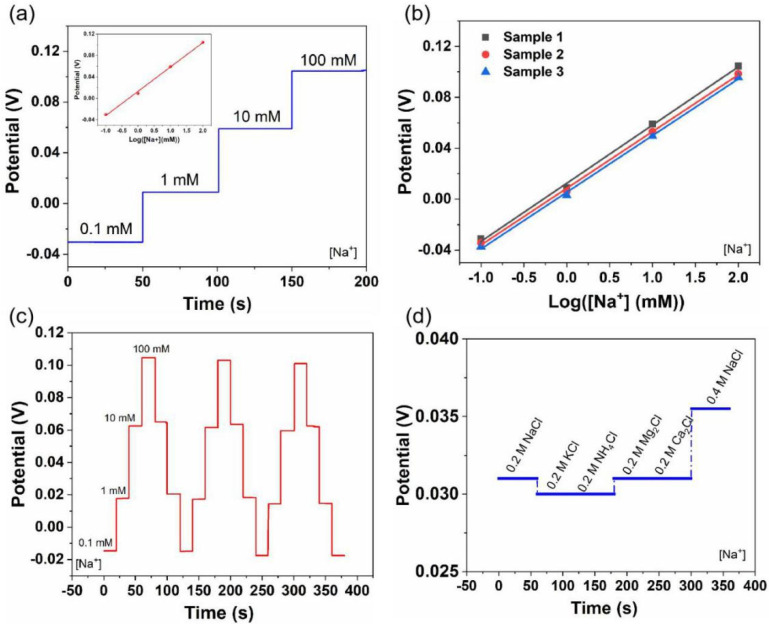
Na^+^-sensing performance of flexible LIG-based sensors: (**a**) sensitivity, (**b**) reproducibility, (**c**) stability, and (**d**) selectivity. Inset in (**a**) is the corresponding calibration plot.

**Figure 4:**
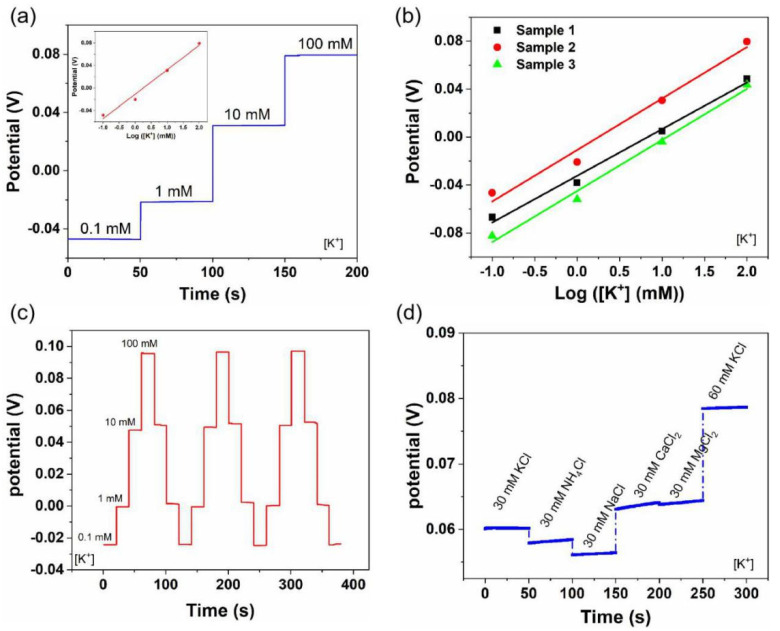
K^+^-sensing performance of flexible LIG-based sensors: (**a**) sensitivity, (**b**) reproducibility, (**c**) stability, and (**d**) selectivity. Inset in (**a**) is the corresponding calibration plot.

Similar to the pH sensors, the Na^+^- and K^+^-sensing performances were also evaluated (Figure 3 and Figure 4). Generally, the sweat contains Na^+^ and K^+^ in the range of 66.3 ± 46.0 mM and 9.0 ± 4.8 mM, respectively [45]. Therefore, we tested the Na^+^- and K^+^-sensing performances of the LIG-based sensors in the electrolyte solutions with target concentrations of 0.1–100 mM. As shown in Figure 3a, the OCP increased linearly with the Na^+^ concentration, and a sensitivity of 45.4 mV/decade was obtained. Meanwhile, the K^+^ sensors showed a similar sensitivity of 43.3 mV/decade (Figure 4a). Furthermore, Na^+^ and K^+^ sensors exhibited good reproducibility, with an average sensitivity of 44.93 mV/decade (RSD = 1.5%) and 41.5 mV/decade (RSD = 5.3%) (Figure 3b and Figure 4b), respectively. Additionally, both sensors showed good reversibility and stability in three-cycle repeated measurements: RSD_Na_ = 0.8%, RSD_K_ = 0.6% (Figure 3c and Figure 4c). Figure 3d and Figure 4d illustrate the selectivity evaluation of Na^+^ and K^+^ sensors in the presence of possible interfering ions at physiologically relevant concentrations. The target electrolytes exhibited negligible interference to the response of each sensor. 

Furthermore, in order to demonstrate that LIG-based sensors are able to withstand mechanical deformation during daily human wear and physical exercise, the pH-, Na^+^-, and K^+^-sensing performances of flexible LIG-based sensors were investigated by monitoring the OCP responses after mechanical bending (radius of curvature is 2 cm). As shown in Figure S2, no apparent variation in the potential response was observed under normal and bent states, indicating the robustness and reliability of LIG-based sensors. Furthermore, as shown in Table S2, we compared the sensing performances of recently reported sweat sensors with the proposed sensor in this study in terms of detection method, sensitivity, linear range, skin wearability, and integration level. Several comments should be noted in future studies: (i) Electrochemical detection (e.g., OCP) is still a mainstream method for the inorganic ions in sweat. (ii) Multiparameter measurement is essential to ensure the accurate evaluation of the health status of individuals. (iii) If a wearable sweat detection system needs to be made small enough for daily use, the smartphone is a valuable supplement.

**Figure 5:**
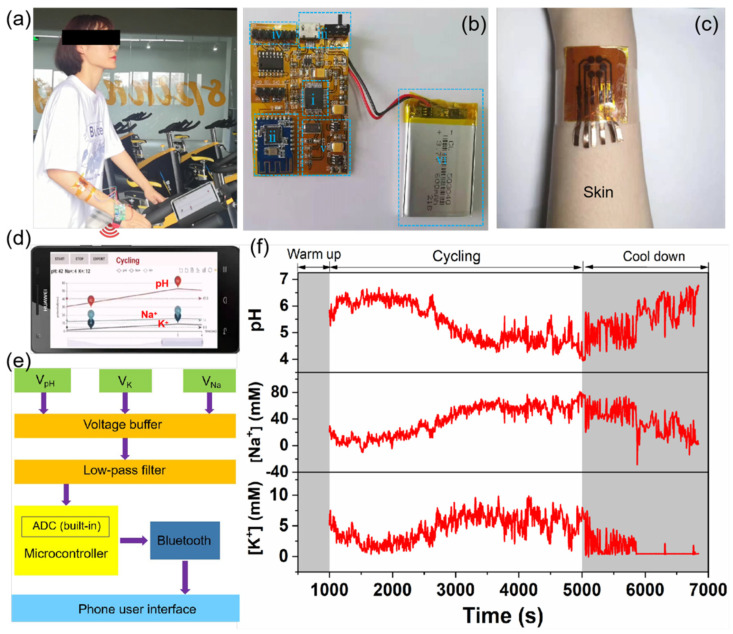
On-body real-time monitoring of sweat during stationary cycling: (**a**) photograph of a LIG-based sensor attached to the forearm of a subject for continuous monitoring of pH, Na^+^, and K^+^ levels in sweat, and the data were collected with a flexible, printed circuit board (FPCB) and then transmitted wirelessly to the smartphone-based application via Bluetooth; (**b**) photograph of the FPCB, which consists of (i) microcontroller, (ii) Bluetooth module, (iii) power switch and charging USB, (iv) sensor connector, (v) power management module, and (vi) lithium-ion battery; (**c**) enlarged photograph of the LIG-based sensor attached to the forearm of a human subject; (**d**) photograph of the developed Android application; (**e**) block diagram of FPCB showing the signal transduction from the LIG-based sensor to the custom-developed mobile application; (**f**) real-time sweat analysis results of pH, Na^+^, and K^+^ concentrations using the integrated wearable sensing system.

To assess the on-body sweat sensor, a LIG-based sensor was attached to a subject’s forearm and integrated with a flexible printed circuit board while they rode a cycling machine (Figure 5a). Figure 5b shows the photograph of the flexible printed circuit board (FPCB), which consists of (i) a microcontroller, (ii) a Bluetooth module, (iii) a power switch and charging USB, (iv) a sensor connector, (v) a power management module, and (vi) a lithium-ion battery. Based on these modules, the FPCB can realize various functions, including signal transduction, conditioning, processing, and wireless transmission. The corresponding block diagram of FPCB is depicted in Figure 5e. A high-impedance voltage buffer was used to measure the voltage difference between Ag/AgCl reference electrodes and ion-selective electrodes (pH, Na^+^, and K^+^). A low pass filter was used to filter the high-frequency noise and stable the output voltage. These voltage analog signals were then converted to digital signals using a 12-bit analog-to-digital converter (ADC) built-in microcontroller. Finally, the data were transmitted to a smartphone via Bluetooth and displayed on a customized Android application (Figure 5d).

Figure 5f shows the profiles of on-body sweat electrolytes as a function of exercise time. Initially, there was no signal response during the first 1000 s because sweat was not generated enough. After 1000 s of biking activity, stable electrochemical signals could be observed. The measured signals were further converted into analyte concentrations using the standard calibration plots obtained by a wearable multiplexed sensing system (Figure S3). From 1000 to 2500 s, sweat pH maintained a stable value of 6.3 and then decreased gradually to a stable value of 4.5. At the stage of cooling down (5000–6800 s), sweat pH increased again, possibly reflecting a common phenomenon that more perspiration would be released from sweat glands when the muscles are relaxed. The Na^+^ and K^+^ sensors showed opposite trends, compared with the pH profile. The concentrations of Na^+^ and K^+^ increased in the beginning and stabilized after 3000 s. With the prolonged time to the stage of cooling down, both sweat signals of Na^+^ and K^+^ decreased rapidly. The overall trends of the sweat electrolyte profiles were comparable to the profiles observed in previously reported on-body tests [46,47]. Additionally, the video of the on-body real-time testing process can be found in Supporting Information (Video S1). Furthermore, we collected the sweat samples during different exercise times (20, 40, and 60 min) and compared the pH, Na^+^, and K^+^ concentrations obtained via the LIG-based sensors and conventional gold standard techniques (pH meter and ICP-MS). As shown in Table 1, the acceptable difference between the two assay results demonstrates the feasibility of the LIG-based wearable device.

## 4. Conclusions

In summary, we demonstrated a wearable epidermal sensor array for noninvasive multiplexed sweat analysis based on laser-induced 3D porous graphene on PI film. The LIG-based sensors showed good performance, with the sensitivities of 51.5 mV/decade (pH), 45.4 mV/decade (Na^+^), and 43.3 mV/decade (K^+^), and the sensing performance was well-maintained under bent states. Good reproducibility, stability, and selectivity were also observed. Additionally, the LIG-based sensor was integrated with a flexible, printed circuit board and custom-developed Android application for simultaneous in situ real-time monitoring of pH, Na^+^, and K^+^ levels in sweat. The correlation of on-body sweat concentrations derived from the integrated system and gold standard methods (pH meter and ICP-MS) demonstrates its capability for real-time sweat analysis. Compared with the commercial devices, e.g., Horiba C-120, the developed devices have low production costs (about USD 10 each). In particular, relying on the facile, high-efficient, and versatile advantages of the LDW technique, our devices have good scalability, as their application can easily be expanded by adding more physiological signals (glucose, uric acid, tyrosine, etc.) to reflect the health status or clinical needs of individuals.

## Figures and Tables

**Table 1:** Comparison of the sensing performance using the LIG-based sensors and conventional gold standard techniques. Sweat samples 1–3 were collected at 20, 40, and 60 min during on-body tests, respectively.

		Sweat 1 (20 min)	Sweat 2 (40 min)	Sweat 3 (60 min)
pH	LIG-based sensor	6.3 ± 0.14	5.6 ± 0.22	4.5 ± 0.18
	pH meter	6.4 ± 0.10	5.3 ± 0.10	4.4 ± 0.10
Na^+^ (mM)	LIG-based sensor	4.16 ± 0.12	31.22 ± 0.15	59.35 ± 0.22
	ICP-MS	3.82 ± 0.06	28.81 ± 0.08	53.06 ± 0.13
K^+^ (mM)	LIG-based sensor	4.85 ± 0.15	4.87 ± 0.21	5.37 ± 0.24
	ICP-MS	5.03 ± 0.10	5.00 ± 0.08	5.28 ± 0.12

## Data Availability

Not applicable.

## Supplementary Materials

The following supporting information can be downloaded at: https://www.mdpi.com/article/10.3390/bios12060397/s1, Figure S1: LED bulb lighted by LIG, Figure S2: pH-, Na^+^-, and K^+^-sensing performance under normal and bent states, Figure S3: pH-, Na^+^-, and K^+^-sensing performance tested on the custom-developed wearable multiplexed sensing system, Table S1: The patterning of 2 × 2 cm squares on PI substrate, Table S2: Comparison of sensing performances, Video S1: On-body real-time testing process. References [48,49,50,51,52,53,54,55,56] are cited in the supplementary materials.
