# Supplementary material for: Laser-Induced Graphene-Based Wearable Epidermal Ion-Selective Sensors for Noninvasive Multiplexed Sweat Analysis
Source: Biosensors (Basel). 2022 Jun 9;12(6):397. doi: 10.3390/bios12060397 (PMC9221044; doi:10.3390/bios12060397)
Supplement: Supplementary file 1 [file biosensors-12-00397-s001.zip › biosensors-1752178-supplementary.pdf]

biosensors

Article

Laser-Induced Graphene-Based Wearable Epidermal Ion-Selective Sensors for Noninvasive Multiplexed Sweat Analysis

Jianjun Liao [1], Xiangya Zhang [1], Zihan Sun [1], Hande Chen [2], Jian Fu [2], Hewei Si [3], Chengjun Ge [1] and Shiwei Lin [3,*]

[1] Key Laboratory of Agro-Forestry Environmental Processes and Ecological Regulation of Hainan Province, School of Ecological and Environmental Sciences, Hainan University, Haikou 570228, China; liaojianjun008@hainu.edu.cn (J.L.); xiangya94264@gmail.com (X.Z.); zihansun266@gmail.com (Z.S.); 990963@hainanu.edu.cn (C.G.)

[2] Hainan Unican Science and Technology Innovation Institute, Haikou 571152, China; chenhande53@gmail.com (H.C.); fujian199702@gmail.com (J.F.)

[3] School of Materials Science and Engineering, Hainan University, Haikou 570228, China; linsw@hainu.edu.cn

* Correspondence: linsw@hainu.edu.cn

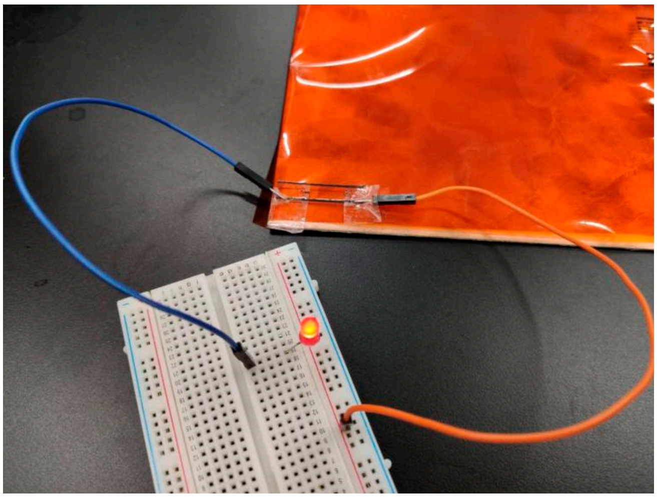

Figure S1. A LED bulb could be readily lighted through a LIG conductive wire, indicating good electrical conductivity of LIG.

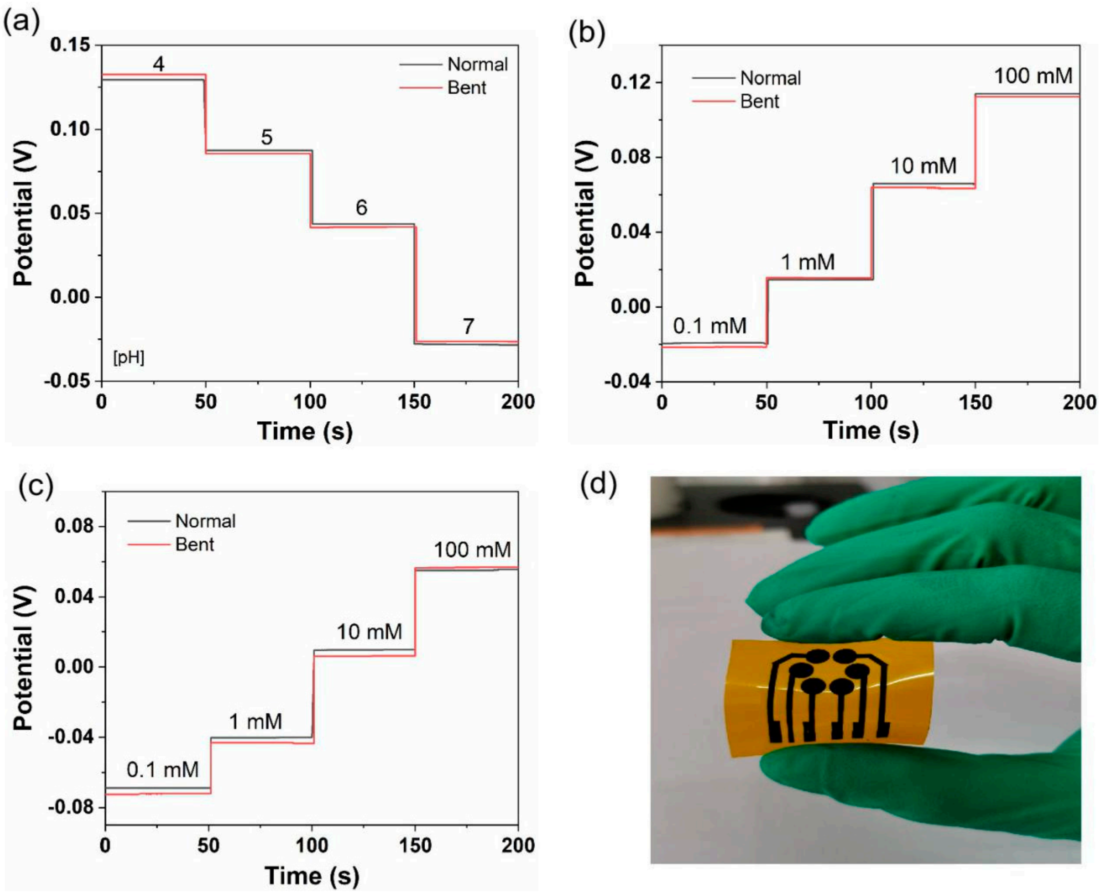

Figure S2. (a) pH, (b) Na^+, and (c) K^+ sensing performance of flexible LIG-based sensors under normal and bent states.

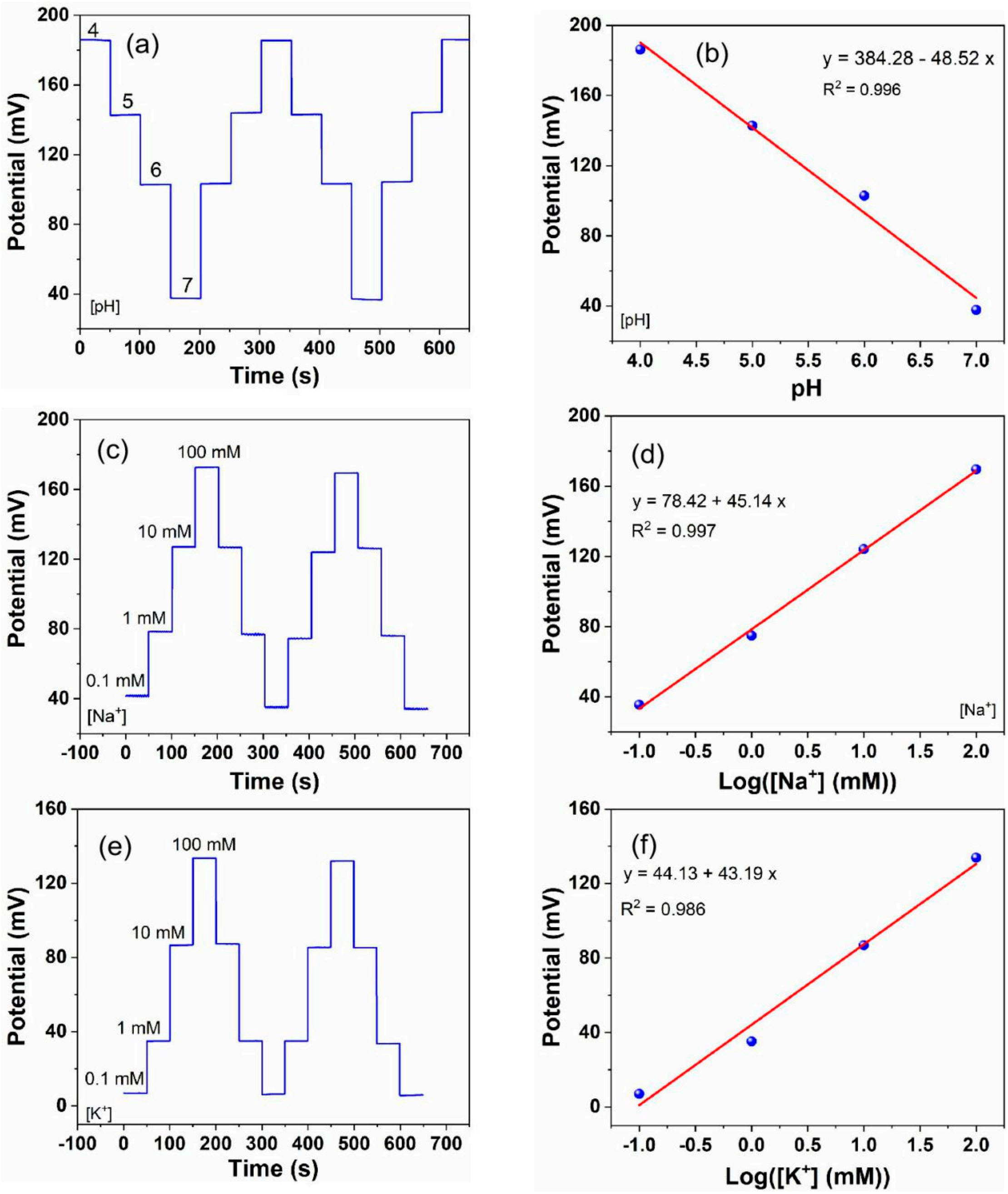

Figure S3. (a,b) pH, (c,d) Na^+, and (e,f) K^+ sensing performance of flexible LIG-based sensors tested on the custom-developed wearable multiplexed sensing system.

Table S1. The patterning of 2 × 2 cm squares on PI substrate was tested with different laser speed and power values.

Laser scribed graphene optimiza-tion		Max. Laser Power (12W)				
		50% (6W)	55% (6.6W)	60% (7.2W)	65% (7.8W)	70% (8.4W)
Laser speed (20 IPS)	10%	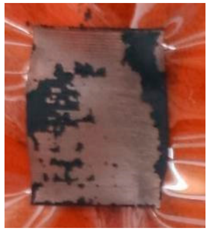	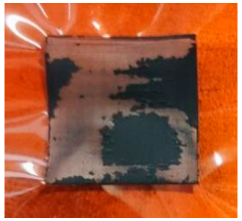	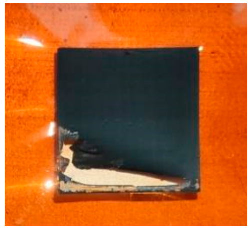	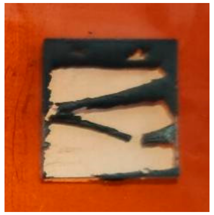	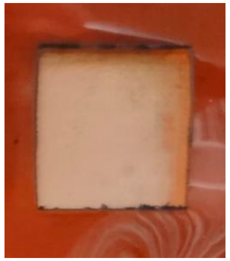
	15%	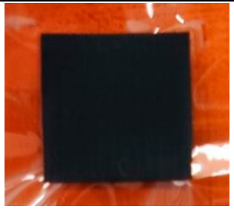 (22 Ω/square)	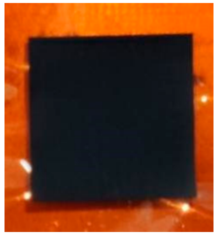 (25 Ω/square)	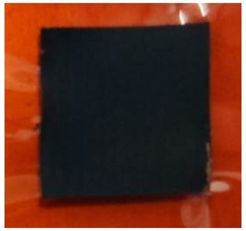 (23 Ω/square)	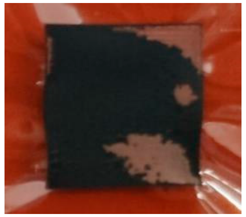	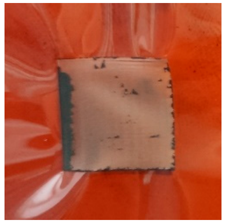

	20%	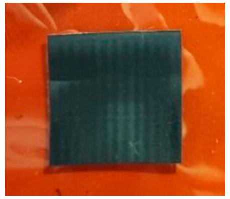 (30 Ω/square)	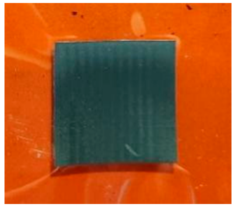 (28 Ω/square)	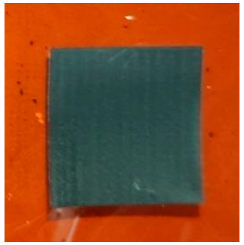 (28 Ω/square)	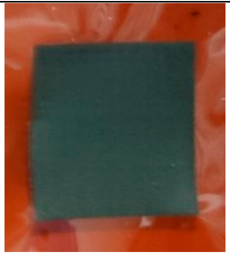 (27 Ω/square)	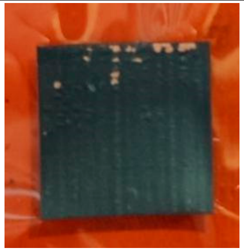 (33 Ω/square)

Table S2. Comparison of the sensing performances of the recently reported sweat sensors with this work.

Year/Reference	Detection method	Sensitivity	Linear range	Skin wearability	Integration level	Communication mode
2021 [1]	OCP	pH: 62.5 mV/decade	pH: 4-8	Yes	No	No
		Na^+: 64.4 mV/decade	Na^+: 10-160 mM			
		K^+: 65.5 mV/decade	K^+: 2-32 mM			
2018 [2]	OCP	pH: --	pH: 4-7	Yes	Yes	Bluetooth
		Na^+: 45.8 mV/decade	Na^+: 10-160 mM		☑Circuit	
		K^+: 35.9 mV/decade	K^+: 2-32 mM		☑Smartphone	
2021 [3]	OCP	Na^+: 50.3 mV/decade	Na^+: 0.1-10 mM	NO	NO	NO

2018 [4]	OCP	K^+: 53.5 mV/decade<br>pH: 71.44 mV/decade	K^+: 0.1-10 mM<br>pH: 4-8	Yes	Yes<br>☑Circuit<br>☑Smartphone	Bluetooth
2014 [5]	OCP	Na^+:63.75 mV/decade	Na^+: 0.1-100mM	Yes	Yes<br>☑Circuit<br>☑PC	Bluetooth
2016 [6]	OCP	Na^+:64.2 mV/decade<br>K^+: 61.3 mV/decade	Na^+: 10-160mM<br>K^+: 1-32mM	Yes	Yes<br>☑Circuit<br>☑Smartphone	Bluetooth
2020 [7]	OCP	Na^+: 56.4 mV/decade<br>K^+: 54.3 mV/decade	Na^+: 0.0001-10mM<br>K^+: 0.0001-10mM	Yes	Yes<br>☑Circuit<br>☑Laptop	Bluetooth
2021 [8]	OCP	pH: 79 mV/decade	pH: 3-8	NO	Yes	NO
2021 [9]	OCP	Na^+: 55.5 mV/decade	Na^+: 1-1000mM	Yes	Yes	Bluetooth

biosensors MDPI

					☑Circuit	
					☑Smartphone	
**This work**	**OCP**	**pH: 51.5 mV/decade**	**pH: 4-7**	**Yes**	**Yes**	**Bluetooth**
		**Na^+: 45.4 mV/decade**	**Na^+: 0.1-100mM**		**☑Circuit**	
		**K^+: 43.3 mV/decade**	**K^+: 0.1-100mM**		**☑Smartphone**	

References

[1] B. Paul, S. Demuru, C. Lafaye, M. Saubade, D. Briand, Printed Iontophoretic-Integrated Wearable Microfluidic Sweat-Sensing Patch for On-Demand Point-Of-Care Sweat Analysis, Adv. Mater. Technol. 2021, 2000910.

[2] L. Wang, L. Wang, Y. Zhang, J. Pan, S. Li, X. Sun, et al., Weaving Sensing Fibers into Electrochemical Fabric for Real-Time Health Monitoring, Adv. Funct. Mater. 2018, 28, 1804456.

[3] F. Wang, Y. Liu, M. Zhang, F. Zhang, P. He, Home Detection Technique for Na^+ and K^+ in Urine Using a Self-Calibrated all-Solid-State Ion-Selective Electrode Array Based on Polystyrene-Au Ion-Sensing Nanocomposites, Anal. Chem. 2021, 93, 8318-8325.

[4] S.Y. Oh, S.Y. Hong, Y.R. Jeong, J. Yun, H. Park, S.W. Jin, et al., Skin-Attachable, Stretchable Electrochemical Sweat Sensor for Glucose and pH Detection, ACS Appl. Mater. Interfaces 2018,10, 13729-13740.

[5] A.J. Bandodkar, D. Molinnus, O. Mirza, T. Guinovart, J.R. Windmiller, G. Valdés-Ramírez, et al., Epidermal tattoo potentiometric sodium sensors with wireless signal transduction for continuous non-invasive sweat monitoring, Biosens. Bioelectron. 2014, 54, 603-609.

[6] W. Gao, S. Emaminejad, H.Y.Y. Nyein, S. Challa, K. Chen, A. Peck, et al., Fully integrated wearable sensor arrays for multiplexed in situ perspiration analysis, Nature 2016, 529, 509-514.

[7] P. Pirovano, M. Dorrian, A. Shinde, A. Donohoe, A.J. Brady, N.M. Moyna, et al., A wearable sensor for the detection of sodium and potassium in human sweat during exercise, Talanta 2020, 219, 121145.

[8] V. Mazzaracchio, L. Fiore, S. Nappi, G. Marrocco, F. Arduini, Medium-distance affordable, flexible and wireless epidermal sensor for pH monitoring in sweat, Talanta 2021, 222, 121502.

[9] H.-R. Lim, Y. Lee, K.A. Jones, Y.-T. Kwon, S. Kwon, M. Mahmood, et al., All-in-one, wireless, fully flexible sodium sensor system with integrated Au/CNT/Au nanocomposites, Sens. Actuators, B 2021, 331,129416.
